# Prognostic significance of chromosome arm 1q gain and methylation class in molecularly defined diffuse leptomeningeal glioneuronal tumor

**DOI:** 10.1007/s00401-022-02507-3

**Published:** 2022-09-29

**Authors:** Jason Chiang, Daniel C. Moreira, Xiaoyu Li, Larissa V. Furtado

**Affiliations:** 1 Department of Pathology, St. Jude Children’s Research Hospital, MS 250, Room C5024A, 262 Danny Thomas Place, Memphis, TN 38105-3678 USA; 2 Department of Global Pediatric Medicine, St. Jude Children’s Research Hospital, Memphis, TN 38105-3678 USA; 3 Department of Oncology, St. Jude Children’s Research Hospital, Memphis, TN 38105-3678 USA

Diffuse leptomeningeal glioneuronal tumor (DLGNT) is characterized by chromosome 1p loss and frequent *KIAA1549::BRAF* fusion [1–3]. DLGNT comprises two methylation classes (MCs): MC-1 and MC-2. Patients with MC-2 DLGNT show inferior progression-free (PFS) and overall survival (OS) [3]. We also reported chromosome 1q gain, seen in all MC-2 DLGNT and 35% MC-1 DLGNT [3], as an adverse prognosticator for PFS and OS in DLGNT [1]. It remains uncertain whether methylation class and 1q status are covariates in DLGNT; hence the outcomes of DLGNT in MC-1 with 1q gain, two seemingly counteracting factors, need to be explored. Moreover, data to assign WHO grades to DLGNT subtypes are limited. Considering its rarity, comprehensively analyzing outcome data in molecularly characterized DLGNT is crucial. We herein present descriptive, Kaplan–Meier, and multivariable Cox regression outcome analyses in a large cohort of molecularly defined DLGNT.

We collected data on 1q and methylation class status of 11 new tumors reviewed at St. Jude Children’s Research Hospital (St. Jude) and additional 21 reported cases for which outcome, copy number variation (CNV), and methylation class information was available [1–3]. The new tumors’ 1q status was confirmed by CNV analysis with the Infinium MethylationEPIC array (850k) and fluorescence in situ hybridization (FISH, when tissue was available) following institutionally established cutoffs for 850k (0.15 using conumee-pipe() in the AnyCN package) and FISH (10%, probe for 1q43) in Clinical Laboratory Improvement Amendments-certified clinical laboratories. The new cases’ methylation class and 1q status were affirmed by Brain Tumor Classifier v.12.5 (https://www.molecularneuropathology.org/mnp/). For cases followed at St. Jude, progression was defined as unequivocal volumetric increases in lesions or leptomeningeal dissemination for initially localized tumors. The status of previously reported cases was updated with current follow-up data.

A total of 32 tumors were included in this study (Supplementary Table 1, online resource). All tumors had 1p loss, and 81.3% (26/32) had *KIAA1549::BRAF* fusion. Single tumors had gene fusions involving *NTRK2/3* or *RAF1*. Chromosome 1q gain was found in 56.3% (18/32) of tumors (Fig. 1a). There was 100% concordance on 1q gain assessment by FISH and 850 k. The 1q gain appeared clonal by FISH, and most tumor cells acquired one copy of 1q. All tumors without and 50% (9/18) of tumors with 1q gain were MC-1. There was no gender predilection for 1q or methylation class. Tumors with and without 1q gain occurred at similar ages (Fig. 1b). Large-scale CNVs other than 1p loss and 1q gain were not recurrent.

**Fig. 1:**
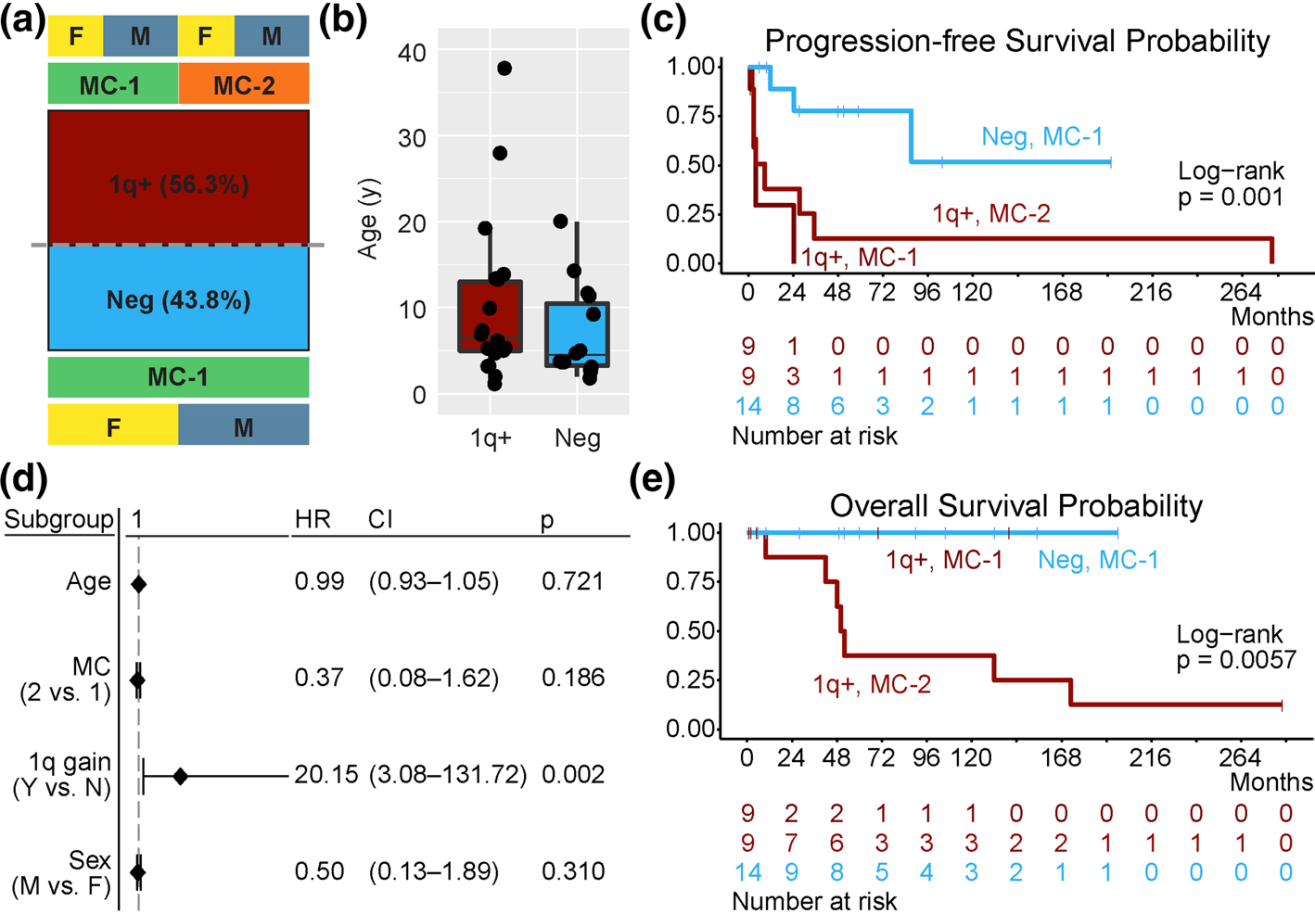
**a** Summary of the study cohort: 56.3% (18/32) of tumors had 1q gain. All tumors in methylation class 2 (MC-2, 9/32) had 1q gain; all tumors without 1q gain (Neg, 14/32) were in methylation class 1 (MC-1). Half of the tumors with 1q gain were in MC-1. There was no gender predilection within the subgroups (*F* female, *M* male). **b** No difference in age at diagnosis (ANOVA, *p* = 0.2839). **c** Kaplan–Meier plot of progression-free survival (PFS) of patients in the three subgroups (MC-1 and no 1q gain, MC-1 with 1q gain, and MC-2 with 1q gain) showed tumors with 1q gain had worse PFS. **d** Multivariable Cox regression analysis with PFS as the endpoint showed that 1q gain is the only significant prognosticator (*HR* hazard ratio, *CI* 95% confidence interval). **e** Kaplan–Meier plot of overall survival (OS) of patients in the three subgroups (MC-1 and no 1q gain, MC-1 with 1q gain, and MC-2 with 1q gain)

Kaplan–Meier analysis of PFS with this expanded cohort confirmed 1q gain was significantly associated with inferior PFS in patients regardless of methylation class (Fig. 1c). Multivariable Cox regression analysis with PFS as the endpoint showed only 1q status significantly correlated with PFS (Fig. 1d). Tumors with 1q gain were approximately 20 times more likely to progress.

Analysis of OS showed MC-2 tumors (all with 1q gain) had inferior survival than MC-1 tumors (with or without 1q gain) (Fig. 1e). Multivariable Cox regression analysis using OS as the endpoint was precluded since deaths have occurred only in patients having MC-2 DLGNT. This might be related to the shorter follow-up of patients with MC-1 tumors with 1q gain (median 2 months, average 24.8 months, range 1–140 months) than MC-2 tumors (median 50 months, average 88.3 months, range 2–286 months). The median time to death for MC-2 tumors was 51 months (average 99.1 months, range 10–173 months), an outcome compatible with WHO grade 3. Longer follow-up of patients with MC-1 tumors with 1q gain is required to ascertain whether mortality rates of patients with MC-1 and MC-2 tumors with 1q gain are comparable. On the other hand, DLGNT without 1q gain showed an outcome compatible with WHO grade 1.

In summary, we demonstrate chromosome 1q gain is the only significant prognosticator affecting PFS in DLGNT regardless of methylation class. Sufficient follow-up is needed for MC-1 tumors with 1q gain to clarify their survival outcomes. Chromosome 1q gain can be detected by FISH along with 1p deletion, the molecular signature of DLGNT, on one tissue section and used as a cost-effective prognosticator. This benefit is most evident in minute specimens, frequent for patients with DLGNT. The underlying mechanism conferring the aggressiveness of DLGNT with 1q gain remains to be investigated.

## Supplementary Information

Below is the link to the electronic supplementary material.

Supplementary file1 (PDF 124 kb)
