# Supplementary material for: Prognostic significance of chromosome arm 1q gain and methylation class in molecularly defined diffuse leptomeningeal glioneuronal tumor
Source: Acta Neuropathol. 2022 Sep 29;144(6):1185–7. doi: 10.1007/s00401-022-02507-3 (PMC9637611; doi:10.1007/s00401-022-02507-3)
Supplement: Supplementary file 1 — Supplementary file1 (PDF 124 kb) [file 401_2022_2507_MOESM1_ESM.pdf]

Age(y)[1]	1q gain	MC	Sex	Addition Molecular Data	Outcome[2]	PFS (m)	Status_PFS	OS (m)	Status_OS	References
3	Neg	1	F	*KIAA1549::BRAF*, 1p loss	SD	28	0	28	0	[1,3]
4	Neg	1	F	*KIAA1549::BRAF*, 1p loss	PD	25	1	155	0	New case
5	Neg	1	F	NTRK2 fusion, 1p loss	SD	10	0	10	0	New case
11	Neg	1	F	*KIAA1549::BRAF*, 1p loss	SD	6	0	6	0	New case
12	Neg	1	F	*KIAA1549::BRAF*, 1p loss	PD	89	1	132	0	[1,3]
14	Neg	1	F	*KIAA1549::BRAF*, 1p loss	SD	106	0	106	0	[1,3]
20	Neg	1	F	*KIAA1549::BRAF*, 1p loss	SD	60	0	60	0	[1,2][3]
2	Neg	1	M	NTRK3 fusion, 1p loss	SD	52	0	52	0	[1,3]
3	Neg	1	M	*KIAA1549::BRAF*, 1p loss	SD	198	0	198	0	[1,3]
3	Neg	1	M	*KIAA1549::BRAF*, 1p loss	PD	12	1	90	0	[1,3]
4	Neg	1	M	*KIAA1549::BRAF*, 1p loss	SD	6	0	6	0	New case
4	Neg	1	M	*KIAA1549::BRAF*, 1p loss	SD	10	0	10	0	New case
5	Neg	1	M	*KIAA1549::BRAF*, 1p loss	SD	49	0	49	0	[1,2,3][3]
9	Neg	1	M	*KIAA1549::BRAF*, 1p loss	SD	10	0	10	0	[1,3]
1	+	1	F	1p loss	SD	1	1	2	0	New case
2	+	1	F	*KIAA1549::BRAF*, 1p loss	SD	1	0	1	0	New case
5	+	1	F	*KIAA1549::BRAF*, 1p loss	SD	1	0	1	0	[1][3]
7	+	1	F	1p loss	SD	1	0	1	0	[1][3]
3	+	1	M	*KIAA1549::BRAF*, 1p loss	PD	3	1	5	0	New case
7	+	1	M	*KIAA1549::BRAF*, 1p loss	PD	4	1	140	0	[1][3]
10	+	1	M	*KIAA1549::BRAF*, 1p loss	PD	1	0	1	0	New case
13	+	1	M	*KIAA1549::BRAF*, 1p loss	PD	25	1	70	0	[1,2,3][3]
19	+	1	M	*KIAA1549::BRAF*, 1p loss	SD	2	0	2	0	New case
5	+	2	F	*KIAA1549::BRAF*, 1p loss	Death	9	1	173	1	[1,3]
5	+	2	F	*KIAA1549::BRAF*, 1p loss	Death	28	1	50	1	[1,3]
6	+	2	F	*TRIMM33::RAF1*, 1p loss	Death	3	1	52	1	[1,3]
14	+	2	F	*KIAA1549::BRAF*, 1p loss	SD	2	0	2	0	[1,2,3][3]
3	+	2	M	*KIAA1549::BRAF*, 1p loss	Death	2	1	42	1	New case
5	+	2	M	*KIAA1549::BRAF*, 1p loss	PD	286	1	286	0	[1,3]
13	+	2	M	*KIAA1549::BRAF*, 1p loss	Death	3	1	48	1	[1,3]
28	+	2	M	1p loss	Death	4	1	10	1	[1,3]
38	+	2	M	*KIAA1549::BRAF*, 1p loss	Death	36	1	132	1	[1,3]

[1]Age at diagnosis (years), [2]PD: progression of disease, SD: stable disease, [3]With updated information
